# Reviewing hereditary connective tissue disorders: Proposals of harmonic medicolegal assessments

**DOI:** 10.1007/s00414-024-03290-4

**Published:** 2024-07-15

**Authors:** Nicola Galante, Maria Francesca Bedeschi, Benedetta Beltrami, Paolo Bailo, Laura Alicia Silva Palomino, Andrea Piccinini

**Affiliations:** 1 Section of Legal Medicine of Milan, University of Milan, Via Luigi Mangiagalli 37, 20133 Milan, Italy; 2 Department of Biomedical Sciences for Health, University of Milan, Via Luigi Mangiagalli 37, 20133 Milan, Italy; 3 Fondazione IRCCS Ca’ Granda Ospedale Maggiore Policlinico, Medical Genetic Unit, Milan, Italy

**Keywords:** Sudden death, Aortic aneurysm/dissection, Child abuse, Clinical forensic medicine, Variants of unknown significance (VUS), Genetic counselling

## Abstract

Hereditary connective tissue disorders (HCTDs) are a heterogeneous group of inherited diseases. These disorders show genetic mutations with loss of function of primary components of connective tissue, such as collagen and elastic fibers. There are more than 200 conditions that involve hereditary connective tissue disorders, while the most known are Marfan syndrome, Osteogenesis Imperfecta, and Ehlers-Danlos syndromes. These disorders need continuous updates, multidisciplinary skills, and specific methodologic evaluations sharing many medicolegal issues. Marfan syndrome and Ehlers-Danlos syndromes show a high risk of early sudden death. As a consequence of this, postmortem genetic testing can identify novel genotype–phenotype correlations which help the clinicians to assess personalized cardiovascular screening programs among the ill subjects. Genetic testing is also essential to identify children suffering from Osteogenesis Imperfecta, especially when a physical abuse is clinically suspected. However, this is a well-known clinical problem even though there are still challenges to interpret genetic data and variants of unknown significance due to the current extensive use of new genetic/genomic techniques. Additionally, the more significant applications and complexities of genomic testing raise novel responsibilities on the clinicians, geneticists, and forensic practitioners as well, increasing potential liability and medical malpractice claims. This systematic review provides a detailed overview on how multidisciplinary skills belonging to clinicians, medicolegal consultants, radiologists, and geneticists can cooperate to manage HCTDs from autopsy or clinical findings to genetic testing. Thus, technical aspects need to be addressed to the medicolegal community since there is no consensus works or guidelines which specifically discuss these issues.

## Introduction

In this systematic review, the authors discuss the medicolegal implications of the three major hereditary connective tissue diseases (HCTDs): Marfan syndrome (MFS), Osteogenesis Imperfecta (OI), and Ehlers-Danlos syndromes (EDS). The medicolegal consultants own a unique background which make them a sort of *bridge* between the individual (dead or living) and the interests of public health. Therefore, technical aspects of HCTDs need to be addressed to the medicolegal community since there is no consensus works or guidelines which specifically focus on these issues. This review aims to provide a detailed knowledge on the medicolegal implications of these disorders, which include the description of postmortem findings and the assessment of current available recommendations on genetic testing from autopsy to cases of living subjects suspected of child abuse. Additionally, it highlights peculiar issues concerning sudden deaths in young athletes suffering from MFS, the importance of correctly addressing the informed consent for genetic testing in HCTDs, the medicolegal significance of VUS (genetic variants of unknown significance – not pathogenetic) and prenatal diagnosis of severe OI.

Marfan syndrome (MIM # 154,700) is one of the most well-recognized hereditary connective tissue disorders, which was named by Antoine-Bernard Marfan, a French paediatrician who first described the condition in 1896. The estimated prevalence ranges from 1:5,000 up to 1:10,000 individuals in the general population [1–3]. Clinical data provide evidence that MFS is primarily an autosomal dominant condition, whereas about 25–30% of the mutations are sporadic and not inherited, resulting from the period of zygote formation [4, 5]. In addition, according to literature, there are few numbers of MFS families with a reported autosomal recessive inheritance model [6]. MFS is caused by mutations in the *FBN1* gene (MIM * 134,797) located on chromosome 15q21.1 [7], encoding for fibrillin-1 which is a large structural macromolecule that contributes to the integrity and function of several connective tissues.

Osteogenesis Imperfecta, also known as Brittle Bone Disease, is a phenotypically and genetically heterogeneous group of heritable connective tissue disorders [8, 9]. Its various types occur in approximately 1 in 15,000–20000 newborns [10, 11]. The individuals affected by OI have low bone mass and bone fragility, a high frequency of fractures, vertebral compressions, long bone deformities, and growth deficiency. Secondary clinical features associated with OI may include blue sclerae, which is the most peculiar feature of this syndrome, dentinogenesis imperfecta (characterized by dentin dysplasia, which results in weak and discolored teeth), limb deformities, hearing loss, reduced respiratory function, cardiac valvular regurgitation, pectus carinatum, clinodactyly, and scoliosis [11, 12]. Most patients affected by OI have an autosomal dominant mutation in *COL1A1* (MIM * 120,150; located on chromosome 17q21.33) or *COL1A2* (MIM * 120,160; located on chromosome 7q21.3) genes, which encode the α1(I) and α2(I) chains of type I collagen.

The term Ehlers Danlos Syndrome (EDS) defines a group of heterogeneous inherited conditions of the connective tissue. The prevalence of EDS is estimated to be about 1:5000 individuals [13]. The most recent classification of EDS proposes a list of major and minor criteria for diagnosing the different subtypes of the condition. It is based on clinical phenotypes, recognizing 13 subtypes, either autosomal dominant or autosomal recessive. Type I EDS is the Classic Ehlers Danlos Syndrome (MIM # 130,000). In over 90% of cases, it is associated with heterozygous mutations in *COL5A1* (MIM *120,215; located on chromosome 9q34.3) or *COL5A2* (MIM *120,190; located on chromosome 2q32.3) genes; less commonly individuals suffering from EDS type I show unusual heterozygous mutations in *COL1A1* gene [14].

## Material and methods

A retrospective systematic review of casualty data was conducted, selecting paper titles and abstracts based on relevance. Literature research was carried out in the most common electronic databases (PubMed, Scopus, Medline and Web of Science) using the following combination of free text protocols, individually and randomly combined through the Boolean operator “AND”: “Marfan syndrome”, “Marfan-like syndromes”, “Osteogenesis Imperfecta”, “Ehlers-Danlos syndromes”, “genotype–phenotype correlations”, “sudden death”, “unexpected death”, “forensic”, “autopsy”, “physical child abuse”, “neglect”, “genetic testing”, “medical liability”, “prenatal diagnosis”, “informed consent”.

**Fig. 1:**
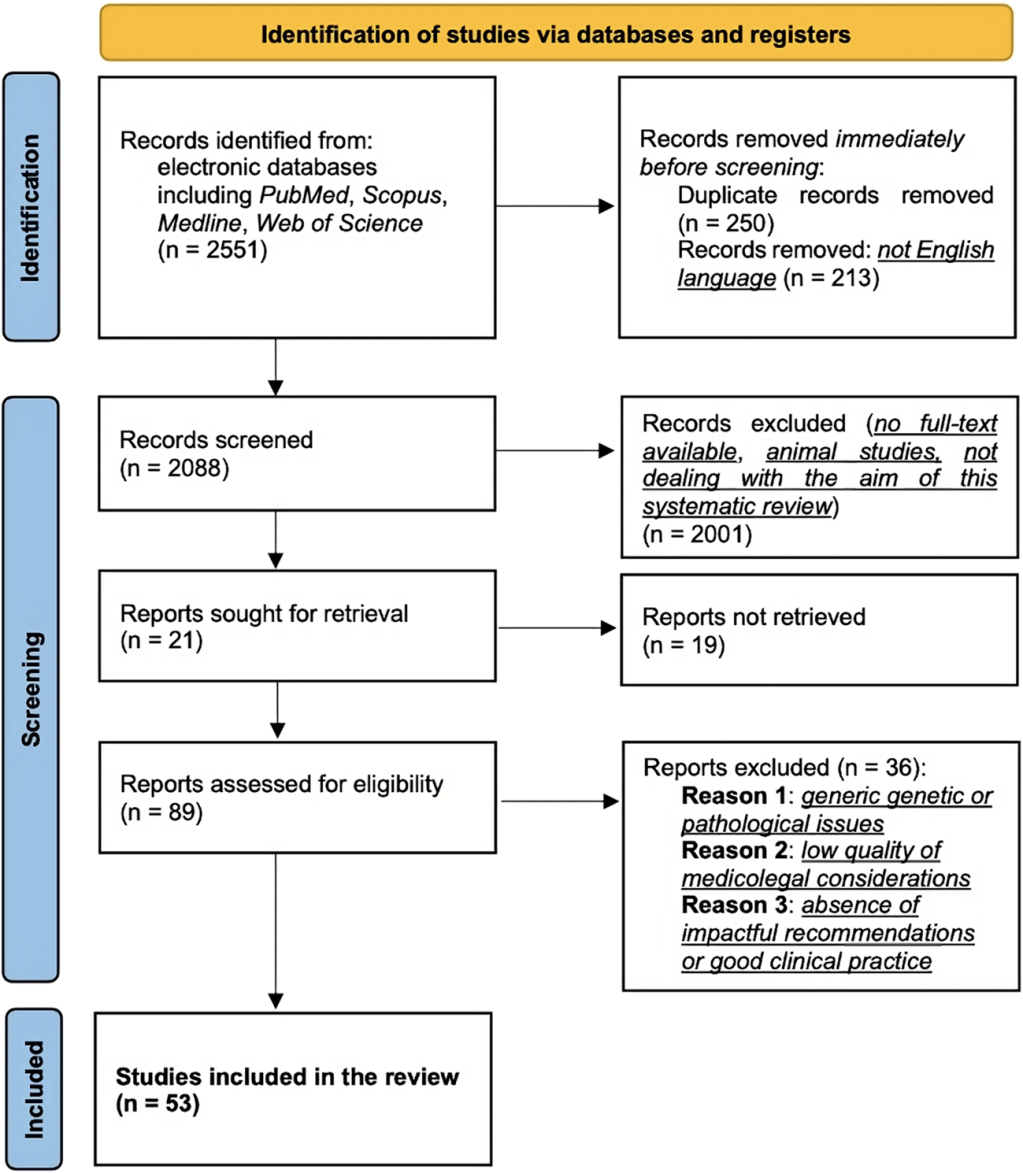
Graphic illustration of the systematic review process based on the PRISMA 2020 statements

The review process is based on the PRISMA 2020 statements [15], which are graphically reported in Fig. 1. The review process included all papers that have been published up to June 2023. Preference was given to recently published papers, but commonly referenced and highly regarded older publications were included. References of selected papers were reviewed for other relevant papers. Only papers fulfilling the following inclusion criteria were included (one at least):

- Sudden and unexpected deaths due to a hereditary connective tissue disorder (HCTDs);
- Description of postmortem findings related to such disorders;
- Usefulness of antemortem and postmortem genetic testing in such conditions;
- Differential diagnosis of physical child abuse in Osteogenesis Imperfecta and Ehlers-Danlos syndrome;
- Discussion of medicolegal issues concerning the significance of genetic testing results, including the significance of VUS, the importance of the informed consent, or the role of prenatal diagnosis.

## Results

**Fig. 2:**
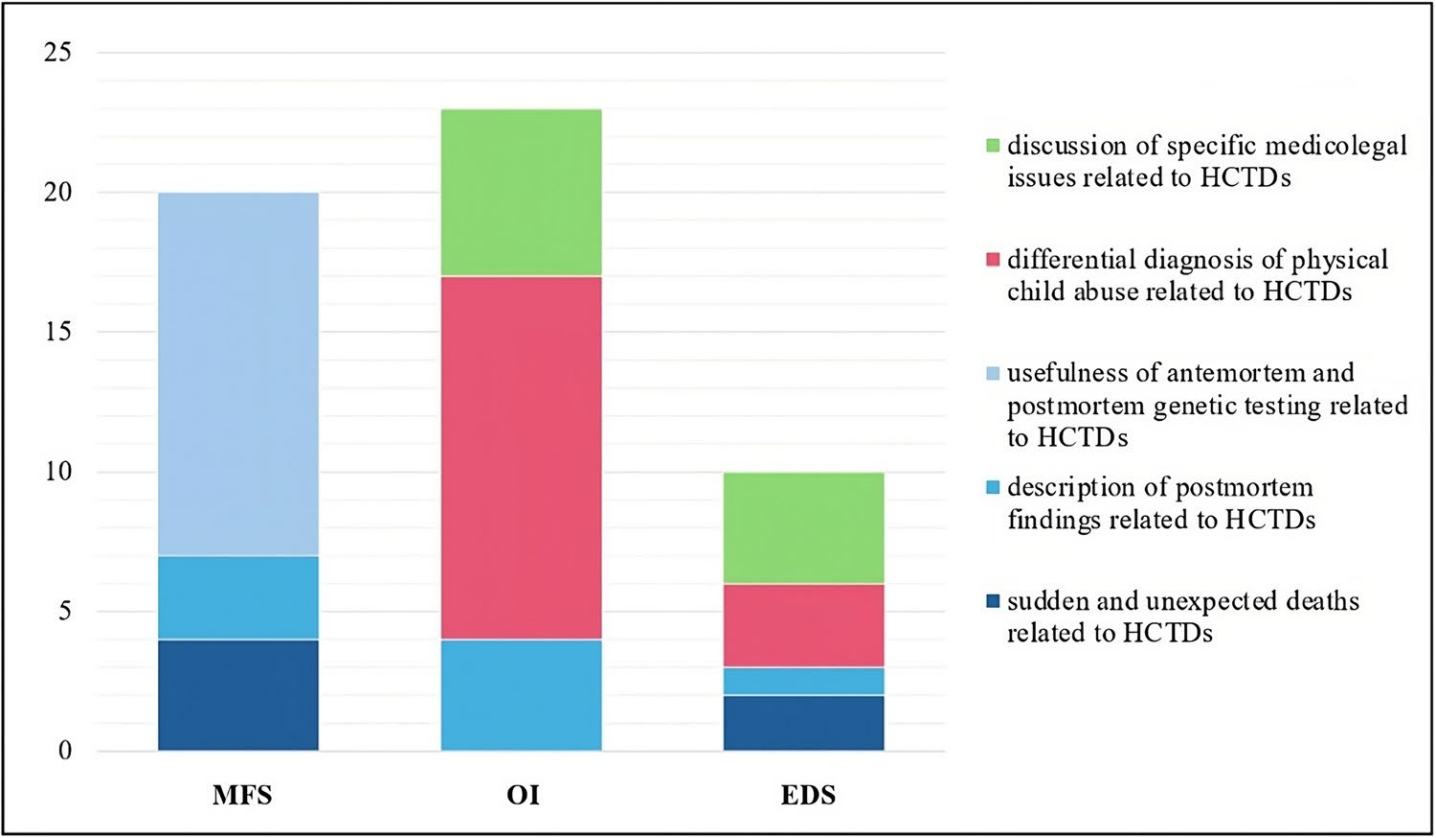
Number of papers selected per inclusion criteria and divided per connective tissue disorder

The literature search provided 53 papers fulfilling the inclusion criteria (one at least), as shown in Fig. 1. Specifically, there were 20 papers dealing with MFS, 23 with OI, and 10 with EDS. However, the literature review showed very few papers which deeply focused on the forensic and medicolegal issues of such disorders. The oldest paper was published in 1989 by C.R. Paterson & S.J. McAllion, dealing with the differential diagnosis between child abuse and Osteogenesis Imperfecta. Figure 2 reports the number of papers which have been selected per inclusion criteria.

The following discussion is divided into 3 major sections (“Marfan syndrome and Marfan-like syndromes”; “Osteogenesis imperfecta”; “Ehlers-Danlos syndromes”) each of which contains subparagraphs discussing specific topics and providing a technical and detailed analysis on the medicolegal issues of such disorders.

## Discussion

### Marfan and Marfan-like syndromes

#### The approach of pathologists upon MFS and MFS-like syndromes

Clinical features and the major autopsy key points about MFS are shown in Table 1 [16–24].

**Table 1:** This table summarizes the major clinical and forensic features upon MFS; furthermore, the genotype–phenotype correlations of specific medicolegal relevance are reported

	Clinical features	Genotype–phenotype correlations of special medicolegal relevance	Forensic features
MARFAN SYNDROME	- Pectus excavatum/chest asymmetry; - Tall habitus with long limbs and fingers; - Hindfoot deformity or plain flat foot arachnodactyly; - Mitral valve prolapse - Bicuspid aortic valve; - Severe scoliosis or thoracolumbar kyphosis; - Skin striae; - Specific facial features (e.g., dolichocephaly, malar hypoplasia, retrognathia)	**- Mutations in exons 24–32 gene *FBN1*** are associated to neonatal mortality and severe MFS phenotype. Such mutations are significantly related to low life expectancy (death < 40 years of age); **- Mutations that cause haploinsufficiency** show a higher risk of cardiovascular deaths rather than dominant negative ones; **- Phenotype severity much depends on truncating and splicing mutations** rather than missense variants	**- Spontaneous aortic dissection or ruptured aneurysms especially among young individuals**; **- Upon histology**: intimal and medial degeneration resulting from the accumulation of mucin pools with fragmentation and altered aggregation of elastic fibers and smooth muscle cells nuclei loss. Van Gieson’s and Masson’s stain enhances disarrangement and variations in length of elastic fibers. Mucin deposits are highlighted by Alcian blue histochemical staining; **- Collect and store generous samples for future genetic investigation** (*see flowchart 1*); - Clinicians should pay attention when evaluating young individuals for sport practice suspected or suffering from MFS

The pathologist who finds a spontaneous aortic dissection or aneurysms rupture especially in young individuals (< 60 years of age) needs to consider an underlying collagen-related diseases. Therefore, the practitioners should adopt a standardized postmortem protocol based on the collection of samples which can allow them to diagnose such disorders. Specifically, a full body forensic autopsy with an in-depth examination of aorta (Letulle method) including carotids, subclavian arteries, celiac artery, mesenteric, renal, and iliac arteries is recommended [25]. By adapting the recommendations of Sabatasso et al. [26], fixation and preservation of the whole aorta is preferable since it can be later examined by an expert cardiovascular pathologist. However, if retention of the whole aorta is not possible for any reasons, full thickness aortic samples should be taken from at least six different points (aortic annulus, ascending aorta, aortic arch, descending aorta, and abdominal aorta supra- and infrarenal). This approach is highly advisable since there may be local variations in the extent of the intimal and medial degeneration [26, 27]. According to de Boer et al. [25], comprehensive histological investigations with special staining methods (as reported in Table 1) of major organs, aorta, and major aortic branches are necessary to verify morphologically the presence of pathological alterations which can be therefore referred to HCTDs.

In these cases, the pathologists need to collect and store generous samples for future genetic analyses (i.e., blood → if absent psoas muscle, spleen, or kidney) [28]. If autopsy or extensive sampling for histological investigations cannot be performed, postmortem radiology may provide a feasible alternative to study the aorta, some of its major branches, and other organs [25]; accordingly, genetic samples could be taken from skin or nails [29, 30].

There are some MFS-like syndromes which share the high risk of sudden deaths related to spontaneous severe aortic events but bare different gene mutations. Among these, Loeys-Dietz syndrome (MIM # 609,192) shows mutations in other genes which are involved in the same pathway of FBN1 and include *TGFBR1* (MIM * 190,181), *TGFBR2* (MIM * 190,182), *SMAD3* (MIM * 603,109), *TGFB2* (MIM * 190,220), *TGFB3* (MIM * 190,230) and *SMAD2* (MIM * 601,366) [23, 25]. Clinical features such as cleft palate, bifid uvula, hypertelorism, and craniosynostosis are suggestive of Loeys-Dietz syndrome rather than MFS. Furthermore, Familial Thoracic Aortic aneurysm and dissection (FTAAD) and its variant with bicuspid aortic valve lack of marfanoid skeletal features, involve different genes (*ACTA2* (MIM * 102,620), *MYLK* (MIM * 600,922), *PRKG1* (MIM * 176,894), *MYH11* (MIM * 160,745), *MFAP5* (MIM * 601,103), *MAT2A* (MIM * 601,468) but show aortic enlargement and early onset of rupture by dissection or aneurysm formation [31], which is also more challenging for pathologists.

Still, a standardized postmortem protocol for spontaneous major aortic events in young individuals is recommended to guarantee equal diagnostic chances among living family members.

Finally, a very careful postmortem examination is needed in the event of a traumatic aortic or major arteries rupture/dissection among individuals suspected or suffering from MFS or MFS-like syndromes. Extensive photographic documentation and sampling with necessary histological investigations (lesion vitality) are required to assess the traumatic mechanism of death.

#### Known genotype–phenotype correlations of MFS

The relationship between sudden death and aortic wall rupture has been identified by several studies [32–39]. Although known *FBN1* mutations are spread throughout the gene, very few genotype–phenotype correlations exist in *FBN1*-related Marfan syndrome. The major existing correlations are presented in Table 1.

A recent review by Stengl et al. [39] reported exhaustively the evidence of strong genotype–phenotype correlations in MFS, highlighting the importance of improving the risk stratification of severe aortic events (TAADs) among family members. Nowadays, only few widely recognized correlations exist [39–48]. Regarding the cardiovascular features, it has been documented that the missense mutations substituting a cysteine had a higher probability of ascending aortic dilation and mitral valve prolapse than mutations creating a cysteine [44]. Thus, not all missense mutations present the same risk of cardiovascular fatalities, and also raise the possibility that variants eliminating cysteine may cause malignant cardiovascular phenotype [40, 46, 47]. Such evidence may indeed suggest a crucial role of this amino acid in the protein structure of fibrillin-1.

#### Recommendations to postmortem genetic testing in MFS and MFS-like syndromes

Recently, the HTAD (Heritable Thoracic Aortic Diseases) Rare Disease Working Group have reported clinical criteria which identify cardiovascular conditions to be investigated with genetic testing [49].

By adapting these criteria in a pathological context (both clinical and forensic), we propose that postmortem genetic testing should be done in the following situations:

i) Adults < 60 years with postmortem evidence of thoracic aortic aneurysms at any level and with a z-score > 2.5 (the z-score indicates the number of standard deviations above the mean);
ii) Any adults with postmortem evidence of thoracic aortic aneurysms at any level and with a z-score > 2 and clinical features suggestive of MFS or MFS-like syndromes;
iii) Children or young adults with postmortem evidence of thoracic aortic aneurysms at any level.

In cases where known pathogenic variants are found, then family members should be contacted to undergo genetic cascade screening.

Gago-Dìaz et al. [50] stressed the recommendation of postmortem genetic testing in sudden death cases due to thoracic aortic dissection. The authors first tried to demonstrate that incorporating the molecular diagnosis via massive parallel sequencing in TAAD autopsy cases is beneficial since these cases remain largely unexplored in the forensic field; among 17 cases, the authors identified two pathogenic variants, two likely-pathogenic ones, and six VUS. Although there are papers which focus on the importance of a molecular approach for sudden cardiac deaths among family members [51, 52], the research by Gago-Dìaz et al. (2017) was the first which analyzed its application on TAADs-related deaths from a pathological to a clinical-genetic setting [50].

However, this approach includes some disadvantages. The interpretation of VUS is more difficult when dealing with a frequently asymptomatic disease with an incomplete penetrance and variable expression – such as Marfan syndrome – which means that not all carriers of the causal mutation develop the clinical manifestations and that a wide range of clinical manifestations exists. To avoid issues of medicolegal liability and assess the best clinical management, Gago-Dìaz et al. proposed a practical way of proceeding [50]: ***a)*** identification of a putative causal mutation → ***b)*** support causality through in silico predictions of pathogenicity; → ***c)*** cascade screening; → ***d)*** functional studies so that to offer the segregation analysis to every willing family member.

This methodology provides one of the strongest types of evidence of causation, but it is unlikely to be feasible in a routine pathological context and this situation opens to the second problematic issue. Gene sequencing is seldom performed worldwide because of the lack of standard procedures existing for postmortem genetic analyses, high costs and necessary permission by the public prosecutor [35]. Thus, many prosecutors and pathologists currently consider a dissected aortic aneurysm as a definitive cause of death, without inquiring further into any underlying genetic disorders [36]. This *status quo* might miss the opportunity to screen at-risk relatives for hereditary diseases and establish appropriate preventative measures prior to complications.

**Fig. 3:**
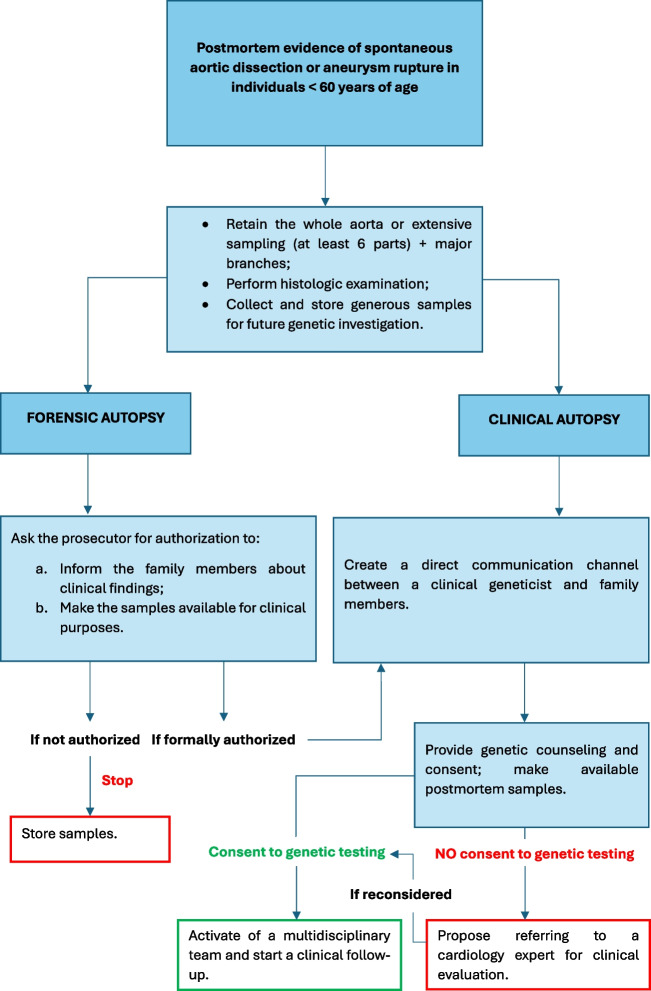
Proposal of a feasible methodology which can be applied in routine practice and depicted in the flowchart 1 for the evaluation of MFS

According to Fellmann et al. [28], we propose a feasible methodology which can be applied in routine practice and depicted in the flowchart 1 (Fig. 3). Since it may take too long for the availability of the test results, family members can empirically start undergoing a precautionary cardiovascular follow-up.

#### MFS and sports in a medicolegal setting

Important medicolegal claims could be raised among sudden deaths or severe aortic events of young adults suffering from MFS or Marfan-like disorders who were screened for athletic competitions and sport. [53]. In such cases, clinicians should follow specific recommendations for the detection of a possible cardiovascular history, which include key questions about exercise-related symptoms, a history of murmur or increased blood pressure, and a family history of premature death or disability from cardiovascular disease in a relative less than 50 years of age [54]. In addition, the Councils on Clinical Cardiology and Cardiovascular Disease in the Young recommend questions aiming to specific knowledge about the occurrence of Marfan syndrome [18]. Noteworthy, in an analysis of 134 athletes who died of cardiovascular causes, 115 athletes were found to have had a standard physical evaluation [55]. Of these, only four (3%) were suspected of having a cardiovascular disease, whereas one athlete (0.9%) was found to have a dilated aorta with a postmortem diagnosis of Marfan syndrome.

Therefore, clinicians need to report accurately anamnestic data and physical examination findings, knowing that further exams such as the echocardiography or the cardiac magnetic resonance are mandatory in the event of any cardiovascular abnormality (MFS is frequently associated to mitral valve prolapse) [56]. They do not necessarily provide significant results due to atypical variants with not specific physical features and without strong genotype–phenotype correlations but represent the best clinical practice to follow which is now available.

### Osteogenesis Imperfecta

#### OI in clinical forensic medicine

Clinical and genetic features about OI are shown in Table 2 [10–12, 57, 58].

**Table 2:** This table summarizes the major clinical, genetic and radiologic features upon OI; furthermore, radiologic differences between OI and NAI are compared and contrasted

	Clinical features and classification INCDS	Genetic features	Radiological features in OI versus NAI, and other forensic features
OSTEOGENESIS IMPERFECTA	1. **Type I**: mildest form with blue sclarae but no bone deformities; 2. **Type II**: extremely severe and perinatally lethal; 3. **Type III**: the most severe form observed in individuals who survive the neonatal period, comprises severe progressive bone deformities and an extremely short stature; 4. **Type IV**: mild to moderate bone deformities, short stature, and normal sclerae; 5. **Type V**: it shows calcification of the interosseous membrane and is both radiologically and phenotypically different from the other types	- Approximately 90% of individuals with OI are heterozygous for mutations in the *COL1A1* and *COL1A2* genes, with dominant pattern of inheritance or sporadic mutations; - Mutations of *IFITM5* gene are associated with OI type V; - In the remaining 10% cases, the disease is correlated to mutations that cause recessive OI in genes such as *FKBP10, LEPRE1, PLOD2, PPIB, SERPINH1, SP7, TMEM38, BCRTAP, BMP1, WNT1, CREB3L1, SPARC, TENT5A*	- **OI may include** apophyseal avulsion fracture, “popcorn” calcifications, intra-osseous calcifications found in the knee metaphyseal and epiphyseal regions, osteopenia on the skeletal survey, long bone diaphysis fractures, Wormian bones, multiple thoracolumbar compression fractures, and L5 spondylolysis; - **NAI may include** symmetric rib fractures, specifically posterior medial and bilateral, complex skull fracture, metaphyseal lesions, scapular fracture, sternal fracture, and spinous process fracture; - **Perform accurate familiar anamnesis, full blood analyses, skin and ophthalmologic examination, samples for DNA analysis** (*see flowchart 2*)

In clinical forensic medicine, child abuse should be evaluated by a multidisciplinary team, including pediatricians, radiologists, medicolegal consultants, geneticists, psychologists, and social workers [59, 60]. In this setting, it is crucial to collect anamnestic data regarding family members, and to perform blood test analyses, including complete blood cell count, serum calcium, phosphorus, alkaline phosphatase concentrations, and serum 25-hydroxy-vitamin D concentration in order to early identify potential metabolic disorders [61]. Therefore, physical examination shows a pivotal clinical role: a thorough skin exam looking for bruises and burns is indeed important when differentiating between OI and NAI (non-accidental injury) [62, 63]. Hence, children with bruises located on the back, ears, and genital area are more indicative of NAI [64]. Furthermore, from a forensic point of view, bruises showing markedly different patterns, shapes, and colors cannot be referred to the same traumatic episode.

The major radiologic features in OI and NAI are summarized in Table 2 [64–70].

Kleinman [71] differentiated among fractures that appear to be:

- *Highly likely* to have resulted from abuse, including metaphyseal fractures, posterior rib fractures, and scapular, spinous process, and sternal fractures;
- *Moderately likely* to have resulted from abuse, including multiple fractures, especially when bilateral, fractures of different ages, epiphyseal separations, vertebral body fractures and subluxations, digital fractures, and complex skull fractures;
- *With low specificity* for abuse, which include clavicular, long bone shaft, and linear skull fractures.

Kleinman [71] and Ablin et al. [60] argued that the radiologic features of OI differ sufficiently from those of abuse to make the distinction straightforward in most cases. D’Eufemia et al. [67] also reported that rib fracture shows a predictive value of 95% for child abuse, especially in the posteromedial site and when occurring in children with less than three years of age. The characteristic mechanism that needs to be produced is a compression around the chest accompanied by the act of squeezing anteriorly and posteriorly the thorax. This type of fracture is extremely rare in patient with OI, and it occurred generally only in the most severe forms but on lateral sides.

#### Differential diagnosis between OI and NAI

A review by Pandya et al. [68] focused on which types of Osteogeneses Imperfecta were most confused with NAI. Specifically, they documented that from the studies reviewed, 25.5% (44 out of 172) of subjects with OI type IV (MIM **#** 166,220) were confused with NAI; conversely, 13.1% (77 out of 589) of subjects with other OI types were confused with NAI. The authors suggest that the difficulty in differentiating NAI from bone disease is complicated by marked variability in the manifestations of classic findings, as reported in Table 2.

Other essential features suggestive of NAI include intra-abdominal trauma resulting in duodenal, spleen or liver hematomas, traumatic brain/spinal cord injury, and bilateral retinal hemorrhages (both intraretinal and preretinal) [64, 72, 73]. For this reason, an ophthalmologic examination with optical coherence tomography and digital wide-field fundus photography could be beneficial since retinal hemorrhages represent the most common findings of abusive head trauma. Ophthalmologic consultation is recommended preferably within the first 24 h and ideally within 72 h [70]. Other rare ocular findings include retinoschisis, retinal folds, chorioretinal scars, and optic nerve sheath hemorrhages.

Unfortunately, from a medicolegal point of view, the literature which directly compares bone diseases with NAI is sparse, published between the 1980s and 1990s, and does not provide enough scientific evidence to differentiate OI from NAI. Furthermore, the methodology does not provide controls for bias confounding or chance since the papers were mainly descriptive in nature. For these reasons, clinical findings are at high risk of misclassification, where sometimes only the physicians’ own instincts make the diagnosis with potential forensic catastrophic consequences. However, genetic testing (typically molecular genetic testing of *COL1A1/2* and *IFITM5* (MIM * 614,757) genes) could be employed to screen potential victims of physical abuse [74]. Zarate et al. [75] conducted a retrospective study (2016) on 43 children with bone fractures in whom physical abuse was suspected and molecular testing for OI was performed. The research revealed only 2 pathogenic mutations on *COL1A1/2* which were also associated to clinical features. The study was only based on the sequencing of type I collagen genes.

Noteworthy, at this time there are no consensus guidelines that identify the circumstances in which genetic testing for OI is indicated or when clinical and radiological evaluation alone can rule out the diagnosis of OI. As suggested by Pepin et al. [76], testing for OI can be completed by sequencing *COL1A1, COL1A2*, and *IFITM5* simultaneously; if these analyses are negative, performing additional genetic testing to identify deletions or duplications (array-CGH or MLPA test). If these tests do not identify a pathogenic variant, in the absence of a strong clinical suspicion, no further genetic analyses are suggested. Genetic testing for the recessive form of OI is indicated only in the presence of other features, including consanguinity, prenatal or neonatal fractures with moderate to severe bone phenotype, congenital contractures, or specific clinical characteristics [76].

#### Challenges to interpret genetic results in OI

**Fig. 4:**
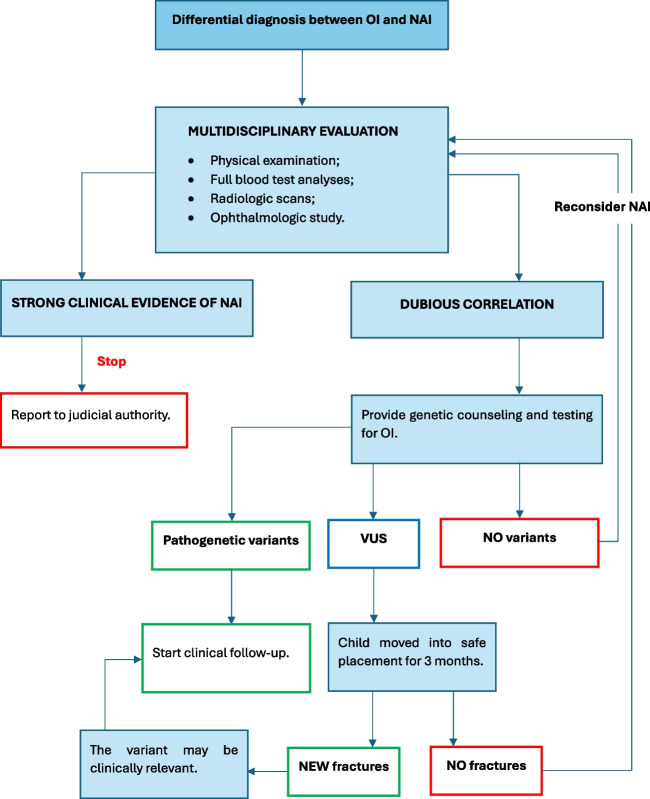
Proposal of a methodological approach to OI and child abuse which is depicted in the flowchart 2

Genetic testing may pose uncertain results reporting variants of unknown significance [75–77]. They are sequence variants of uncertain pathogenicity in genes known to cause inherited Mendelian diseases in which molecular genetic testing has a proven clinical validity and utility. The European Medical Genetics Quality Network’s best practice guidelines for the laboratory analysis of Osteogenesis Imperfecta recommend that VUS should be identified as unclassified until segregation within the family has been investigated and/or functional evidence becomes available [78]. This approach may be hard to carry out due to long-lasting follow-up or other practical issues. A recent study by Canter et al. [79] focused the attention on the clinical significance of VUS during an evaluation for potential child physical abuse, showing important forensic implications. In such conditions, the child with evidence of VUS may be moved into safe placement and then evaluated at 3 months (the outer limit of hard callus appearance), providing the best time window to document potential new or healing fractures [79]. Specifically, evidence of new fractures while the child is in foster care could suggest that there may exist genotype–phenotype correlations [76, 79]. Conversely, the absence of new fractures may indicate that the variant is not clinically relevant and a more diligent handling of the child. Promising future criteria include the possibility of performing proteomic studies to functionally investigate the collagen production, and the extension of genetic testing to the family members [77]. In this regard, OI is a disorder showing high penetrance, even within the same family, so that genetic results should always be compared to the family clinical history [79]. A proposal of a methodological approach to OI and child abuse is depicted in the flowchart 2 (Fig. 4). Finally, the identification of child abuse in individuals suffering from OI is very challenging, resulting in a topic almost totally neglected in the scientific literature [80].

#### OI and prenatal diagnosis in a medicolegal setting

Prenatal diagnosis of OI is the result of the corroboration of many factors which include ultrasonography (US) pathological findings and prenatal specific genetic analysis [81].

Specifically, US examinations may reveal multiple intrauterine fractures, thoracic hypoplasia, bone demineralization, and shortening and deformity of long bones [82]. The more severe pathological features are found, the more OI type II (MIM **#** 166,210) (lethal form) is probable to diagnose. However, it is essential to highlight that prenatal evaluation sometimes leads to expectations that are not confirmed on postnatal evaluation, and that usually this is not automatically the result of a medical malpractice. Thus, as reported by Kidszun et al. [83], this way of differentiating lethal subtype from milder forms of OI is flawed deeply. In fact, OI subtypes were developed by using retrospective data such as radiograph findings, genetics, and clinical course. By these criteria, OI type II was diagnosed when the affected subject had died in utero or shortly after birth. The criteria were not developed to be predictive, and the sensitivity or specificity of different findings as predictors of prognosis has never been scientifically validated – which is also a recurrent issue in clinical forensic medicine with OI. Furthermore, these subtypes were created in 1979 without the consideration of currently available medical interventions which can deeply modify the prognosis.

Genetic testing results are also confounding and difficult to interpret since OI is a highly heterogeneous disorder in which reliable genotype–phenotype correlations are very sparse and contradictory in literature [83–86]. Specifically, quantitative mutations (glycine substitutions and split site) of *COL1A1* and *COL1A2* are mainly responsible for OI type I (MIM **#** 166,200), whereas qualitative defects are significantly associated to OI types II–IV [85, 86]. Prenatal diagnosis and the prognostic value of OI based on US and genetic testing are very difficult because of the large number of skeletal dysplasia, their phenotypic variability, and overlapping features, which must be considered in the event of any medical claims. Overall, parents always need to be counseled very carefully and the limitations of prenatal diagnostic results need to be discussed in detail.

### Ehlers-Danlos syndromes

#### A group of overlapping syndromes between MFS and OI

Clinical, pathological, and genetic features are shown in Table 3 [87–99].

**Table 3:** This table summarizes the major clinical, pathological and genetic features upon EDS

	EDS main subtypes and clinical features	Genetic features	Forensic features
EHLERS-DANLOS SYNDROMES	- **Classic type** (**I/II**); **hypermobile type** (**III**); **vascular type** (**IV**); - Hypermobile joints; - Scoliosis; - Skin laxity and fragility; - Atrophic scars and frequent bruises; - Aneurysm spontaneous rupture; - Hollow organ spontaneous rupture; - Uterine rupture during pregnancy	- ***COL5A1*, *COL5A2*** (α1 and α2) chains of type V collagen; - ***COL3A1*** (α1) chain of collagen III which is associated with EDS type IV - ***COL1A1****, ****COL1A2***, **procollagen N-peptidase** - Mutations are generally autosomal dominant; rarely autosomal recessive	- Multidisciplinary approach in the forensic examination of clinical-pathological features, with photo documentation (*see flowchart 3*)

**Fig. 5:**
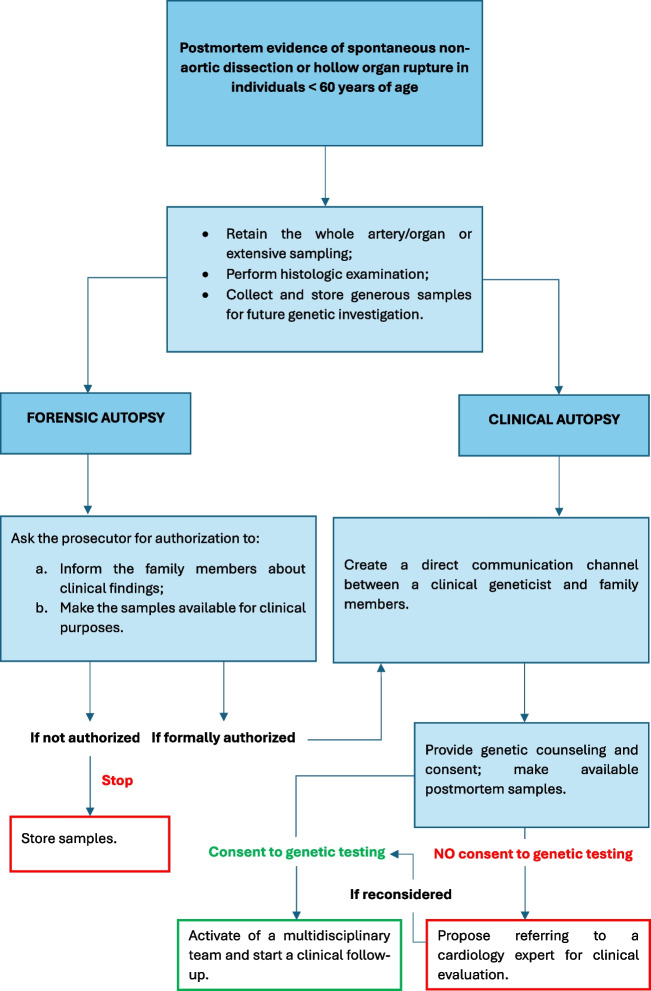
Proposal of a feasible methodology which can be applied in routine practice and depicted in the flowchart 3 for the evaluation of EDS cases

These syndromes are heterogenous and may show overlapping manifestations of MFS and OI with which they share similar medicolegal issues. EDS type IV (MIM * 601,468, vascular-type) shows poor average life expectancy due to spontaneous pneumothorax, pulmonary hemorrhage, aneurysms rupture, arterial dissection, and hollow organ rupture [87–89]. Specifically, major thoracic and abdominal arteries are mostly involved in rupture and dissection [90]. Rupture of the uterus is more frequent within the last stages of pregnancy [91], whereas the sigmoid colon is the intestinal segment most affected by perforation [92]. The postmortem examination should be performed in harmony with the procedural approach that has been given for MFS and MFS-like syndromes (see “The approach of pathologists upon MFS and MFS-like syndromes”)*.* However, this syndrome requires more caution rather than MFS since it may show spontaneous non-aortic artery rupture/dissection [90, 95, 96] and hollow organ rupture. Furthermore, histology findings are often less specific than the other vascular syndromes [90] and may include ***a)*** reduced dermal skin thickness; ***b)*** thin collagen bundles with large interstitial spaces in the reticular dermis; ***c)*** increased thickness of cutaneous elastic fibers; ***d)*** thinning of the adventitia of the aortic wall [98]. For those purposes, the pathologist should collect the whole organ/artery and specimens of skin for the histologic examination with samples for future genetic analyses. A proposal of a feasible methodology which can be applied in routine practice is depicted in the flowchart 3 (Fig. 5).

EDS can be considered in the differential diagnosis of child maltreatment or abuse since injuries due to minor trauma could mimic typical maltreatment injuries. Subjects suffering from EDS have multifaceted skin manifestations due to hyperextensibility and fragility of the skin with increased bruising and atrophic scar formation [97, 99, 100]. Accidental injuries are primarily located over the knees, anterior tibial area, and areas with underlying bony prominences with small and single bruising [101–103]. As reported for OI, the main aim of practitioners in such situations is to differentiate between an accidental trauma versus a NAI [104], which should be evaluated in a multidisciplinary setting.

### The importance of genetic counselling for HCTDs

Genetic counselling has several ethical, legal and social implications. Genetic counselling is usually led by a medical geneticist. An individual affected (or suspected to be affected) by a genetic condition can search for or can be referred by other specialists to a medical geneticist. The medical geneticist, if necessary, along with other health professional in a multidisciplinary team is responsible for collecting the medical history, performing the risk evaluation, suggesting the genetic test, commenting the result of the test and eventually proposing surveillance or interventions.

In the setting of OI, finding a VUS is a highly sensitive and delicate issue. In this case, the geneticist plays a key role in the classification of the variant. It is essential to evaluate and collect a meticulous medical history and to extend the analysis to the parents and, if necessary, the siblings of the child, even though OI has incomplete penetrance and variable expressivity, both intra- and interfamilial. To understand the significance of the variant, it is important to discuss the case with the laboratory that performed the analysis, and consider the possibility of additional testing, such as protein studies or a skin biopsy to evaluate the collagen synthesis. These assessments must be done as quickly as possible to avoid negative legal and psychological consequences for children who are placed in a safe environment for a skeletal assessment as precaution. Another important issue is the difficulty to explain the significance of a VUS to the family and other healthcare professionals, as they may assume that any variant mentioned in a medical report is pathogenic. It is challenging to explain the uncertainty of a VUS. To avoid misunderstanding and communication problems, thorough pre-test counselling is essential [79].

Genetic testing in sudden death is also a highly delicate endeavour. It should require a multidisciplinary team comprising a geneticist and the pathologist that performed the autopsy to communicate with the relatives, ensure accurate information dissemination, collect samples, and to obtain informed consent for potentially extending the analysis [105]. The results of genetic testing can profoundly impact the deceased individual’s family members. If a pathogenic variant is detected in the deceased, first-degree relatives may opt to undergo genetic testing to confirm or rule out the presence of the same variant. Communicating these results poses challenges, necessitating referral of family members to a medical geneticist with expertise in the relevant conditions [106]. In the event of death of an individual suspected of MFS or EDS, where genetic testing in unfeasible, family members must be referred to a medical geneticist. The medical geneticist can evaluate the kin according to Ghent Criteria (for MFS) or the 2017 International Classification of the Ehlers-Danlos Syndrome and, when appropriate, propose genetic testing [14, 21].

Lastly, regarding the costs, in Italy genetic services, such as genetic counselling and genetic testing, are provided to patients and their relatives in a context of Public Health Genomics, when there is a risk of a genetic condition. Thus, when a medical geneticist suspects a genetic disease, genetic tests are offered free to the patients and cascading to the family members [107].

## Conclusive remarks

We propose some conclusive remarks to summarize the major medicolegal issues among HCTDs:

- The postmortem evidence of spontaneous aortic dissection or aneurysm rupture among individuals < 60 years of age should be considered as suspicious of Marfan syndrome or Marfan-like syndrome.
- The postmortem evidence of spontaneous non-aortic dissection, aneurysm or hollow organ ruptures among individuals < 60 years of age should be considered as suspicious of Ehlers-Danlos syndromes.
- Osteogenesis Imperfecta and Ehlers-Danlos syndromes can be considered in the differential diagnosis with child abuse: a multidisciplinary team is highly preferable for the evaluation of such cases.
- Genetic testing may not ensure determining results, especially when dealing with variants of unknown significance (VUS); however, a thorough genetic counselling and a functional clinical approach are fundamental.

## Data Availability

The datasets generated during and/or analyzed during the current study are available from the corresponding author on reasonable request.
